# From Anatomy to Pathology: How Well Can AI Interpret Static Point-of-Care Ultrasound Images?

**DOI:** 10.3390/diagnostics16213647

**Published:** 2026-11-04

**Authors:** Salih Fettahoğlu, Ertuğrul Ak, Süreyya Tuba Fettahoğlu, Adem Az

**Affiliations:** 1 Department of Emergency Medicine, University of Health Sciences, Kanuni Sultan Suleyman Training and Research Hospital, Atakent Mahallesi, Turgut Ozal Boulevard 46/1, Kucukcekmece, Istanbul 34303, Türkiye; salihfettahoglu52@gmail.com; 2 Department of Emergency Medicine, School of Medicine, Istanbul Medipol University, Kavacik Mahallesi, Ekinciler Caddesi No. 19, Beykoz, Istanbul 34810, Türkiye; 3 Department of Emergency Medicine, University of Health Sciences, Haseki Training and Research Hospital, Ugur Mumcu Mahallesi, Belediye Street 7, Sultangazi, Istanbul 34265, Türkiye; kaymaker4303@gmail.com (S.T.F.); adem.aaz@gmail.com (A.A.)

**Keywords:** point-of-care ultrasound, emergency medicine, multimodal large language model, artificial intelligence, image interpretation, acquisition metadata, prompt engineering

## Abstract

**Background/Objectives**: Multimodal artificial intelligence may support point-of-care ultrasound (POCUS) interpretation in emergency medicine, but its performance on static images and sensitivity to acquisition context remain uncertain. This study evaluated GPT-5.6 Sol and assessed whether probe-region information improves interpretation across view/plane, anatomy, and pathology domains. **Methods**: In this paired comparative in silico study, 92 educational ultrasound images from seven anatomical regions were assessed under standardized uninformed (image only) and informed (image plus probe-region code) prompting conditions. Two independent raters scored responses against textbook-based references in three domains: view/plane identification, anatomical structure identification, and pathological finding detection. Each domain was scored from 0 to 2. Disagreements were resolved by a third ultrasound expert, and fully correct responses were defined as domain scores of 2. The primary outcome was the total performance score (0–6). Secondary outcomes included domain-specific scores, fully correct response rates, and exploratory subgroup analyses. Wilcoxon signed-rank and McNemar tests assessed paired scores and fully correct rates, respectively. **Results**: Total performance was higher with informed prompting (median (interquartile range), 5 (4–6) versus 4 (2–5); *p* < 0.001). Interrater agreement was substantial to almost perfect across evaluation domains (quadratic-weighted κ = 0.750–1.000). Fully correct rates increased from 58.7% to 88.0% for view/plane identification and from 47.8% to 82.6% for anatomical structure identification (both *p* < 0.001). Pathological finding detection improved from 35.9% to 48.9% (*p* = 0.002). Among pathology-positive images, missed or incorrectly labeled pathology remained frequent (44.8%) despite informed prompting. Regional differences in total performance were significant only under uninformed prompting (*p* = 0.002). **Conclusions**: Probe-region information substantially improved view/plane and anatomical structure identification, but pathological finding detection remained comparatively weak. In static POCUS interpretation, improved anatomical recognition did not translate into reliable pathology detection. Persistent false-negative and false-positive errors do not support independent diagnostic use. Potential applications in POCUS education and anatomical labeling require further validation.

## 1. Introduction

Large language models (LLMs) have demonstrated notable performance in medicine in recent years. The success of tools such as ChatGPT on medical licensing exams and in text-based clinical vignettes has strengthened the view that these models could serve as supportive instruments in clinical decision-making [1,2,3]. However, clinical practice does not rely solely on text-based information. Diagnostic and therapeutic decisions often require the interpretation of visual data, including radiologic imaging and ultrasonography (US). For a comprehensive assessment of the clinical utility of LLMs, their performance on image-based tasks therefore requires systematic evaluation. Text-based evaluations cannot determine whether a model can directly recognize clinically relevant findings in medical images.

A recent development in this field involves multimodal LLMs (M-LLMs) that can process text and visual inputs jointly [4]. Altinbilek et al. [5] compared task-specific and general-purpose artificial intelligence (AI) models in electrocardiogram (ECG) interpretation and found that a customized model outperformed general-purpose models, highlighting the relevance of task-specific adaptation to visual clinical performance. Studies evaluating GPT-4 on national medical licensing examinations, following earlier ChatGPT assessments, suggest advances in general medical knowledge processing, although these cross-study comparisons are indirect [1,6,7]. However, success on text-based tasks does not necessarily generalize to image interpretation. Image interpretation requires simultaneous spatial awareness, visual pattern recognition, and integration with clinical context [8,9]. Bhayana et al. [3] reported encouraging performance on radiology board-style questions without images; however, that study did not evaluate direct image interpretation. Similarly, Ümit et al. [8] showed in a study on visual ECG interpretation that a customized GPT model still struggled to match the performance of emergency medicine specialists. Doğan et al. [9] also found that a task-specific computer-vision AI model outperformed GPT-based models in the analysis of radiologic images. Collectively, these studies indicate that the reliability of GPT-based models for visual clinical tasks remains uncertain.

Evidence specific to US is more limited. US is a widely used and operator-dependent imaging modality that directly influences bedside decision-making, particularly in emergency medicine and intensive care settings [10,11,12]. Its portability and real-time bedside availability make it particularly valuable for time-sensitive clinical assessments, while scanning-window recognition helps establish the expected anatomical orientation. In point-of-care ultrasound (POCUS), recognizing the scanning window and visible anatomical structures provides the basis for interpreting pathological findings. The probe region is also part of the acquisition context available to the clinician performing the bedside examination. Evaluating whether this limited information improves model interpretation is therefore relevant to the development of AI support for POCUS. Early feasibility studies suggest that M-LLMs may be able to describe certain sonographic features and perform classification tasks, but performance can vary and may be sensitive to prompt design [13,14]. Comparisons with AI-based computer-aided diagnosis (AI-CAD) further suggest that LLM-based approaches may have important limitations, particularly with respect to consistency, verifiability, and achieving clinically meaningful thresholds [13]. However, prompt conditions, scoring approaches, and reporting elements remain heterogeneous across the existing literature, which limits comparability and reproducibility. Differences in prompt wording may alter the contextual information available to the model, while inconsistent scoring criteria and incomplete methodological reporting make independent replication difficult.

Recent studies further emphasize the distinction between direct image interpretation and reasoning from information supplied by clinicians. Chen et al. [15] found that adding clinician-recorded ACR TI-RADS features improved thyroid nodule classification compared with image-only input, although the combined strategy did not outperform features-only input in overall accuracy. This suggests that the improvement may be driven primarily by clinician-supplied features rather than additional information extracted directly from the image. Zou et al. [16] reported that adding key images improved breast ultrasound report auditing but also identified model-specific false-positive outputs and limitations relative to physician-interpreted findings. In pediatric appendicitis, Altintas Mese et al. [17] found that classification performance was driven primarily by structured ultrasound report text, with limited benefit from adding images. These studies evaluated organ-specific diagnostic or reporting tasks using inputs that could contain substantial diagnostic information. The effect of limited acquisition metadata, without descriptions of sonographic findings or suspected diagnoses, warrants separate evaluation across different POCUS regions and interpretation domains.

The aim of this study was to quantitatively evaluate the textbook-referenced image-interpretation performance of GPT-5.6 Sol, an M-LLM, on static US images using standardized prompt conditions and predefined scoring criteria. We additionally examined how providing limited acquisition metadata in the form of a probe-region code influenced performance and characterized residual error patterns that may constrain clinical translation. By separately assessing view/plane identification, anatomical structure identification, and pathological finding detection across seven anatomical regions relevant to POCUS, we examined whether gains in orientation and anatomical recognition were accompanied by comparable improvements in pathological assessment. This paired in silico evaluation addresses a question relevant to emergency medicine POCUS while remaining distinct from validation of real-time bedside performance.

## 2. Materials and Methods

### 2.1. Study Design and Setting

This study was designed as a paired comparative in silico evaluation of the image interpretation performance of GPT-5.6 Sol (hereafter, the model) using educational static US images. No real patient data, biological specimens, clinical records, or personally identifiable information were used. All images were obtained from anonymized educational textbooks that contained no patient identifiers or related metadata [10,11,12]. This approach enabled a controlled evaluation using standardized reference images without accessing identifiable patient data or clinical records. The copyrighted textbook images were used solely for model evaluation and were not redistributed; no original images are reproduced in this manuscript.

Because the study did not involve human participants, identifiable patient information, or clinical intervention, ethics committee approval and informed consent were not applicable. Reporting followed the STARD-AI principles for AI-based diagnostic evaluation studies [18], and LLM-specific methodological elements were structured, where applicable, using the TRIPOD-LLM checklist [19]. We used GPT-5.6 Sol (OpenAI, San Francisco, CA, USA), an LLM with advanced reasoning and multimodal capabilities. Model access was provided between 20 July 2026 and 25 July 2026. The Thinking variant was selected because it is intended to support complex, multi-step tasks through enhanced reasoning. To improve reproducibility and to minimize potential carryover effects from prior conversation context, each static US image was processed in a separate and independent chat session for each prompt condition.

Each ultrasound image was uploaded individually as a JPEG attachment through the chat interface using predefined, unmodified standardized prompt templates (Appendix A). Before each image, a fixed role instruction directed the model to respond as a point-of-care US (POCUS) expert. Two prompt conditions were used: an uninformed condition (image only) and an informed condition providing limited acquisition metadata in the form of a probe region code (e.g., RUQ, LUQ, RLQ, LLQ, SU, SC, PSL, MAX, ANT) without any organ name, clinical history, or suspected diagnosis. All interactions were conducted in English to support standardization of medical terminology and anatomical descriptions.

Model outputs were recorded verbatim as raw text without any post-processing or manual editing. Each output was linked to the corresponding image identifier to create the analysis-ready dataset. When the GPT interface did not allow user configuration of decoding parameters (e.g., temperature and top-p), the platform default settings were used without modification.

### 2.2. Data Source, Image Selection, and Eligibility Criteria

The study dataset consisted of static US images obtained from educational US textbooks (i.e., atlas-style reference images and accompanying explanations) that are commonly used in clinical training [10,11,12]. The selected textbooks are academic/educational resources that provide standard sonographic anatomy, predefined reference scan planes, and representative images.

Image selection was performed without any model training and followed predefined eligibility criteria designed to assess static US interpretation performance. Inclusion criteria were: (i) the image was designated by the textbook as a standard reference view/plane, (ii) target anatomical structures were clearly visible and distinguishable, (iii) the image contained no prominent artifacts or explanatory overlays/markers (e.g., arrows, labels, or text), and (iv) the image had sufficient quality to support interpretation based on a still frame. Images that disrupted anatomical integrity, contained substantial artifacts, or substantially limited interpretability were excluded. These restrictions minimized potential visual confounding and prevented explanatory annotations from revealing the reference findings. The image-selection process involved screening textbook images against predefined eligibility criteria, followed by independent expert verification of reference findings before inclusion.

Selected images were categorized into seven major anatomical groups to reflect the clinical diversity of bedside POCUS in emergency medicine. These categories included cardiac (*n* = 12), thoracic (*n* = 12), abdomen (*n* = 17), inferior vena cava (IVC) and abdominal aorta (*n* = 12), renal and urinary system (*n* = 12), peripheral vascular (*n* = 12), and other examinations (*n* = 15). Each anatomical group included multiple standard scan planes to reflect the diversity of bedside POCUS views and avoid restricting the evaluation to a single canonical plane. The final dataset consisted of 92 independent static images.

All images were extracted from the original PDF documents to include only the relevant anatomical region while preserving image integrity, and they were presented to the model under identical conditions. Although image dimensions varied, all images were standardized after cropping/extraction by enforcing a minimum resolution of 640 × 480 pixels, saved in a consistent file format (high-quality JPEG) without additional compression, while preserving the original grayscale/color information to maintain image fidelity throughout the analysis.

### 2.3. Evaluation and Scoring Process

All model-generated responses were evaluated against predefined reference standards. The reference standard was defined as the descriptions and explanations provided in the corresponding US textbooks. Before model evaluation, the reference anatomical structures and pathological findings were independently verified by two ultrasound experts, and only images with confirmed reference findings were included in the study. For each image, the model was instructed to generate a structured response. The expected components were: (i) identification of the US view/plane, (ii) description of visible anatomical structures, and (iii) identification and brief justification of pathological findings, if present.

To reduce assessment bias, model outputs were coded using image identification numbers, and the anatomical category of each image was masked during scoring. All evaluations were performed by two independent raters who were blinded to each other’s assessments and to the image category; however, they were not blinded to the prompt condition because the model outputs could contain prompt-specific information. The raters assigned scores by adhering to the textbook reference descriptions. No educational feedback, model steering, or re-prompting was applied during the evaluation process. When disagreements occurred, a third US expert served as an adjudicator and made the final decision. All analyses were conducted using these final consensus scores.

Each evaluation domain was rated using an ordinal 0–2 scale. For view/plane accuracy, a score of 2 indicated full concordance with the textbook reference view/plane, a score of 1 indicated partial concordance (e.g., correct orientation with incorrect or incomplete terminology), and a score of 0 indicated an incorrect view/plane identification. For anatomical accuracy, a score of 2 indicated complete and correct identification of the key anatomical structures visible in the image using appropriate terminology (Appendix B), a score of 1 indicated incomplete or partially correct identification, and a score of 0 indicated incorrect or irrelevant anatomical descriptions. For pathological finding detection, a score of 2 indicated correct and justified identification of the pathology when present, and correct recognition that no pathology was present when absent (true negative). In this domain, a score of 1 indicated a partially correct or incomplete description. A score of 0 indicated an incorrect pathological assessment, including missed pathology, incorrect pathology labeling, or identification of pathology when none was present (false positive). For example, correctly recognizing the scanning orientation but incompletely specifying the view was considered partially correct (Score 1). In contrast, reporting pericardial effusion instead of the reference finding of right ventricular dilatation was considered incorrect for pathological finding detection (Score 0; Supplementary Figure S1, Panel C).

For each image, domain scores were summed to generate a total score ranging from 0 to 6. This scoring approach was designed to quantitatively assess the model’s ability to recognize and interpret static US images in accordance with textbook-based reference standards.

### 2.4. Outcomes

The primary outcome was the total performance score under each prompt condition, calculated as the sum of the three domain scores (view/plane identification, anatomical structure identification, and pathological finding detection), ranging from 0 to 6.

Secondary outcomes included domain-specific performance scores for view/plane identification, anatomical structure identification, and pathological finding detection, as well as the proportion of responses rated as fully correct (score = 2) within each domain. Exploratory secondary analyses included comparisons of performance across anatomical regions and pathology-status subgroups (pathology-positive vs. pathology-negative).

### 2.5. Statistical Analysis

All statistical analyses were performed using IBM SPSS Statistics for Windows, version 28.0 (IBM Corp., Armonk, NY, USA). Given the ordinal nature of the scoring system, non-parametric methods were used. Categorical variables are presented as frequency (*n*) and percentage (%), and ordinal scores are summarized as median and interquartile range (IQR). Means were additionally reported to facilitate interpretation of small performance differences. The 95% confidence intervals (CIs) for fully correct response rates were calculated using the Wilson score method.

Interrater reliability between the two independent raters was assessed using quadratic-weighted Cohen’s kappa (κ) for ordinal agreement and interpreted according to Landis and Koch (κ = 0.61–0.80, substantial; κ = 0.81–1.00, almost perfect) [20]. In case of disagreement, a third US expert adjudicated the final score; all main performance analyses were conducted using these consensus scores.

For each US image, domain-specific scores (view/plane accuracy, anatomical structure identification, and pathological finding detection; each scored 0–2) and the total performance score (0–6) obtained under the two prompt conditions (uninformed vs. informed) were compared as paired data using the Wilcoxon signed-rank test. To assess whether performance differed across the three scoring domains within the same prompt condition, domain scores were compared as related samples using the Friedman test; when significant, post hoc pairwise comparisons were performed using Wilcoxon signed-rank tests with Bonferroni correction.

To explore differences in performance across anatomical categories (e.g., cardiac, thoracic, abdominal, etc.), domain and total scores within each prompt condition were compared across independent groups using the Kruskal–Wallis test; when significant, pairwise comparisons were performed using Dunn–Bonferroni post hoc tests.

“Fully correct” responses were defined as outputs receiving a score of 2 in a given domain. Between-prompt differences in fully correct rates for the same images were tested using McNemar’s test after dichotomizing scores as 2 versus <2. For the three domain-specific paired comparisons within each outcome family, statistical significance was assessed using a Bonferroni-adjusted threshold of *p* < 0.0167, while unadjusted p-values were reported. Anatomical-region comparisons were exploratory, and post hoc regional pairwise comparisons were adjusted using the Dunn–Bonferroni method.

All tests were two-sided, and *p* < 0.05 was considered statistically significant.

## 3. Results

### 3.1. Interrater Reliability

Interrater agreement statistics and percent agreement values are summarized in Table 1. The interrater reliability analysis performed on 92 static US images showed high agreement between the two expert raters across all evaluation domains. Under the uninformed condition, the highest agreement was observed for Identification of Anatomical Structures (κ = 0.943, 95% CI 0.883–0.987) and Pathological Finding Detection (κ = 0.943, 95% CI 0.889–0.989). Agreement for View/Plane Identification was substantial (κ = 0.750, 95% CI 0.624–0.863). Under the informed condition, agreement increased across all domains, and perfect agreement was observed for Identification of Anatomical Structures (κ = 1.00).

### 3.2. Overall GPT-5.6 Sol Performance

Table 2 summarizes GPT-5.6 Sol performance across three evaluation domains (view/plane identification, anatomical structure identification, and pathological finding detection) under two prompt conditions on the same images (uninformed vs. informed). The total performance score was significantly higher in the informed condition (median (IQR), 5 (4–6)) than in the uninformed condition (4 (2–5)) (*p* < 0.001). The mean paired increase in total performance score was 1.04 points (95% bootstrap CI, 0.74–1.38).

For view/plane identification, the ordinal score distribution was significantly higher in the informed condition (median (IQR), 2 (2–2)) than in the uninformed condition (2 (1–2)) (*p* < 0.001). The fully correct rate increased from 58.7% (54/92) in the uninformed condition to 88.0% (81/92) in the informed condition (*p* < 0.001). Similarly, for anatomical structure identification, the ordinal score distribution was significantly higher in the informed condition (median (IQR), 2 (2–2)) than in the uninformed condition (1 (1–2)) (*p* < 0.001). The fully correct rate increased from 47.8% (44/92) in the uninformed condition to 82.6% (76/92) in the informed condition (*p* < 0.001). For pathological finding detection, the ordinal score distribution was also significantly higher in the informed condition (median (IQR), 1 (0–2)) than in the uninformed condition (0 (0–2)) (*p* = 0.001). The fully correct rate was 35.9% (33/92) in the uninformed condition and 48.9% (45/92) in the informed condition, and this difference was statistically significant (*p* = 0.002).

### 3.3. GPT-5.6 Sol Performance by Anatomical Region

Table 3 compares the distribution of GPT-5.6 Sol performance across anatomical regions relevant to bedside POCUS in emergency medicine under the uninformed and informed conditions. Under the uninformed condition, the highest mean total scores were observed for Thorax (5.08 ± 0.90) and Abdomen (4.11 ± 2.00), whereas the lowest mean total score was observed in the IVC/Aorta category (2.17 ± 1.95) (*p* = 0.002).

Under the informed condition, score increases were observed across all anatomical categories. The largest numerical increases in total score were observed for Others (+2.00), IVC/Aorta (+1.66), and Vascular (+1.59). In contrast, smaller increases were observed in the Thorax (+0.17) and Cardiac (+0.33) categories. Under the informed condition, total scores did not differ significantly across anatomical regions (*p* = 0.528).

**Figure 1:**
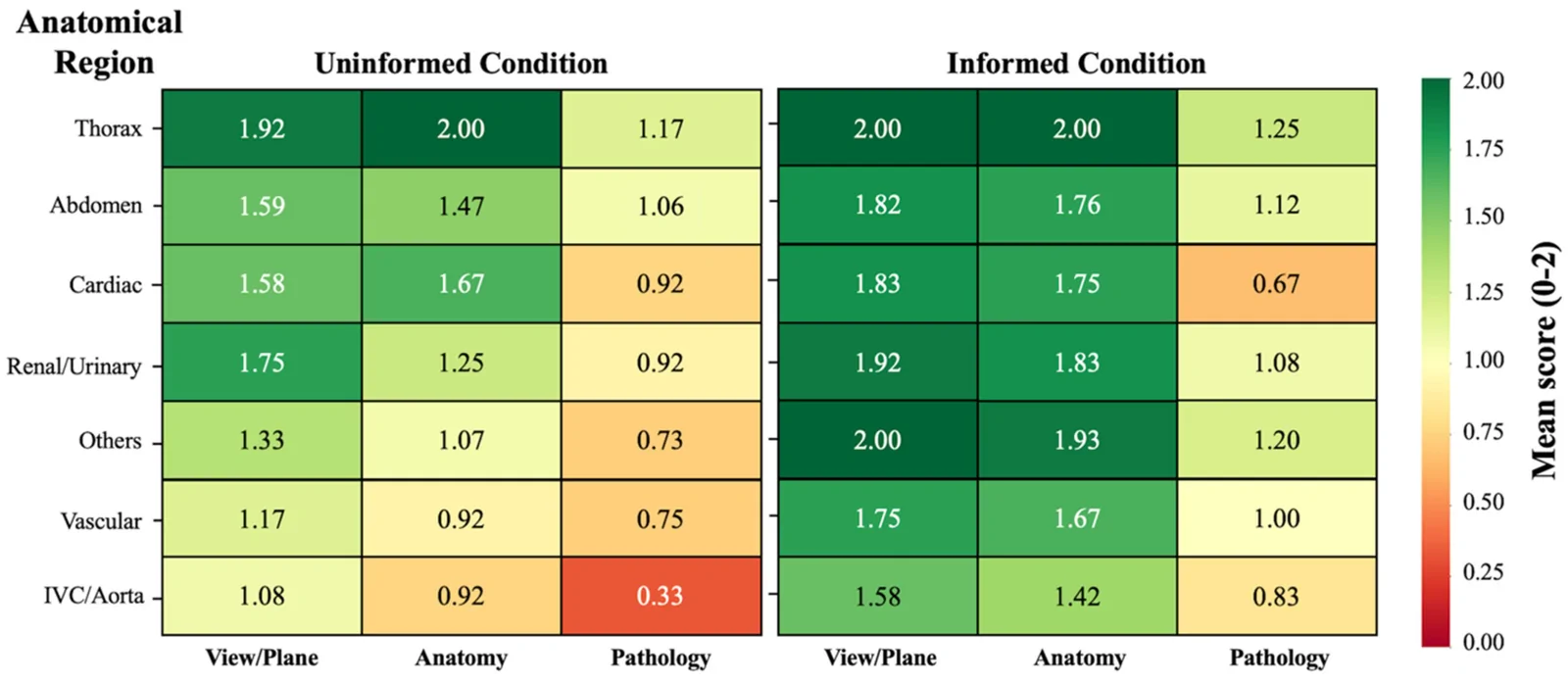
Heatmap of mean domain scores (0–2) of GPT-5.6 Sol across anatomical region categories and evaluation domains. Note: Values represent the mean performance score (range 0–2) for each evaluation domain. Higher scores indicate better performance, while lower scores indicate poorer performance. Abbreviations: IVC, inferior vena cava.

Figure 1 shows the mean domain scores across anatomical regions under the uninformed and informed prompt conditions. Mean scores increased in the informed condition across nearly all regions in the View/Plane and Anatomy domains. Across prompt conditions, the Thorax category had the highest mean total scores. The mean score for Thorax reached 2.00 in both the View/Plane and Anatomy domains. Numerical increases were also observed in the Others category (View/Plane: 1.33 vs. 2.00; Anatomy: 1.07 vs. 1.93), in the IVC/Aorta category for View/Plane (1.08 vs. 1.58) and Anatomy (0.92 vs. 1.42), and in the Vascular category for View/Plane (1.17 vs. 1.75) and Anatomy (0.92 vs. 1.67).

However, mean scores in the Pathology domain remained lower than those in the other domains under both conditions. Numerical increases in the Pathology domain were observed in IVC/Aorta (0.33 vs. 0.83), Others (0.73 vs. 1.20), and Vascular (0.75 vs. 1.00). In contrast, smaller changes were observed in Thorax (1.17 vs. 1.25) and Abdomen (1.06 vs. 1.12). In the Cardiac category, the mean Pathology score was lower in the informed condition than in the uninformed condition (0.92 vs. 0.67). The lowest mean score in the Pathology domain was observed in the IVC/Aorta category under the uninformed condition (0.33).

### 3.4. Pathology Prevalence and Distribution

Of the 92 static US images evaluated, 58 (63.0%) were identified as pathology-positive and 34 (37.0%) as pathology-negative according to the textbook-based reference. The 58 pathology-positive cases were distributed across anatomical categories, with the highest frequency observed in the abdomen (*n* = 14), followed by cardiac (*n* = 9) and other examinations (*n* = 9). The remaining positive findings were distributed among the renal–urinary (*n* = 8), thorax (*n* = 7), vascular (*n* = 6), and IVC–aorta (*n* = 5) categories.

Table 4 summarizes the performance of GPT-5.6 Sol in pathological finding detection stratified by case status (pathology-positive vs. pathology-negative). Among pathology-positive images (*n* = 58), true-positive responses (Score 2) increased from 21/58 (36.2%) under the uninformed condition to 27/58 (46.6%) under the informed condition (+10.4 percentage points), while false-negative responses (Score 0) decreased from 31/58 (53.4%) to 26/58 (44.8%) (−8.6 percentage points). Among pathology-negative images (*n* = 34), true-negative responses (Score 2) increased from 12/34 (35.3%) to 18/34 (52.9%) (+17.6 percentage points), and false-positive responses (Score 0) decreased from 19/34 (55.9%) to 16/34 (47.1%) (−8.8 percentage points).

## 4. Discussion

This paired in silico study evaluated the image-interpretation performance of GPT-5.6 Sol on static US images representing anatomical regions relevant to bedside POCUS in emergency medicine and examined whether providing limited acquisition metadata in the form of a probe-region code improves accuracy. Overall performance increased under informed prompting, with the largest gains observed in view/plane identification and anatomical structure identification, where fully correct rates rose from 58.7% to 88.0% and from 47.8% to 82.6%, respectively. Nevertheless, pathological finding detection improved more modestly and remained the lowest-performing domain, with fully correct responses remaining below 50% even when metadata was provided. GPT-5.6 Sol performance also varied by anatomical region in the uninformed condition, whereas regional differences were no longer statistically significant under informed prompting. Although providing the probe location improved the identification of normal findings, the persistence of a high proportion of responses classified as false negatives (44.8%) and frequent false-positive interpretations highlights an important limitation in the model’s pathological assessment. Our findings suggest that while M-LLMs may support basic orientation and anatomical description on static US frames, improved performance in these tasks should not be equated with reliable pathology detection.

Providing GPT-5.6 Sol with limited acquisition metadata in the form of probe-region information substantially improved performance in view/plane identification and anatomical structure recognition. This pattern is consistent with recent US studies showing that targeted prompting and structured textual guidance can enhance model performance [14,21]. In fetal US, expert-designed custom prompts improved Vision LLM interpretation compared with standard prompting [14], and in breast US, workflows using clinician-generated sonographic descriptions supported BI-RADS classification and malignancy prediction [21]. Similarly, Yao et al. [22] reported that combining breast ultrasound images with report-derived textual information improved GPT-4o diagnostic accuracy from 58% to 70% compared with image-only input. More recently, Chen et al. [15] found that clinician-recorded ACR TI-RADS features improved ChatGPT-5.4 classification of thyroid nodules compared with image-only input, although the features-only strategy achieved the same overall accuracy as the combined image-and-feature strategy. These findings emphasize that the information supplied alongside an image may itself contribute substantially to diagnostic performance. Our study extends this literature by examining a more limited input: probe-region information without organ names, clinical history, suspected diagnoses, or descriptions of sonographic findings. Related image and vision-language approaches have also shown promise in anatomically constrained tasks such as carotid artery segment recognition [23], although these task-specific systems differ from the general-purpose model evaluated here. For future POCUS evaluations, acquisition metadata should therefore be explicitly described, and its contribution to performance should be assessed separately from that of clinical or lesion-specific information. A comparison of the present findings with relevant studies published between 2024 and 2026 is presented in Table 5.

Despite this clear gain in view and anatomy identification, pathology detection was limited, with fully correct performance remaining below 50% even in the informed condition and the proportion of false-negative responses among pathology-positive images remaining high at 44.8%. Our findings indicate that the addition of minimal metadata was not sufficient to overcome the model’s weakness in disease-level interpretation. A similar caution emerges from prior US-focused LLM studies. Brin et al. [24] reported that GPT-4V correctly identified the anatomical region in 60.9% of ultrasound images, whereas pathology identification accuracy was only 9.1%, similarly highlighting the discrepancy between anatomical recognition and pathological interpretation. In thyroid nodule classification, M-LLM performance has been reported as feasible but still imperfect, with concerns regarding discordance from reference outcomes and the risk of clinically consequential misclassification [13,25]. Recent findings in pediatric appendicitis further illustrate this limitation: Altintas Mese et al. [17] found that performance was driven primarily by structured ultrasound report text, while image-only interpretation performed poorly. In breast ultrasound report auditing, Zou et al. [16] reported benefits from adding key images but also identified model-specific false-positive outputs and poorer malignancy classification with key-image input than with physician-interpreted findings. Although these studies differ in their models, tasks, and reference standards, they support the need to distinguish performance based on clinician-supplied information from direct visual interpretation. Our multi-regional evaluation shows that, for the tested model and image set, the limitations in pathology detection persisted despite improved anatomical recognition. These findings do not support independent diagnostic use for the evaluated tasks.

The dissociation between the large improvement in anatomical recognition and the comparatively modest improvement in pathology detection also provides a possible explanation for the observed performance pattern. Probe-location information may reduce ambiguity at the level of global scene interpretation by constraining which organs, structures, and canonical planes are likely to appear in the image. This may explain why performance improved most clearly in tasks involving orientation and structural identification. However, pathology detection requires recognition of subtle echogenic changes, border irregularities, shadowing patterns, small effusions, asymmetry, or other fine-grained semeiologic deviations from normal. Broader discussions of visual integration in medical LLMs have emphasized that multimodal capability should not be equated with robust diagnostic reliability [26]. In our study, GPT-5.6 Sol appeared to benefit from contextual anchoring for macro-anatomic recognition, but this advantage did not translate proportionally into pathology-level interpretation. This explanation remains hypothetical because the study did not examine the model’s internal reasoning processes. Potential applications in view/plane confirmation or anatomical labeling therefore require separate validation rather than being inferred from the improvement in total score alone.

The region-specific analyses further support a task-dependent interpretation of model performance. Although regional differences became less pronounced under the informed condition overall, pathology scores in the cardiac category decreased numerically after probe-location information was provided. In contrast, thoracic, vascular, and IVC/aortic views generally showed numerical improvement with contextual input. In visually and semeiologically complex domains such as cardiac US, one possible but untested explanation is that contextual labeling may encourage reliance on expected anatomical patterns when a static frame incompletely captures the pathological finding. However, the cardiac subgroup was small, and this numerical decrease does not establish that probe-region information adversely affects cardiac interpretation. It should be regarded as an exploratory finding that warrants further investigation. More generally, our results indicate that improved plane recognition should not be assumed to translate into reliable pathological assessment, particularly in examinations where motion and temporal information contribute substantially to interpretation.

These findings are relevant to bedside POCUS in emergency medicine because recognizing the intended scanning window and visible anatomy is only one component of interpreting an examination. The present study evaluates this distinction using static educational images; it does not establish clinical benefit during live scanning. Unlike curated textbook images, real-world bedside POCUS examinations are dynamic and frequently involve suboptimal acoustic windows, patient movement, and imaging artifacts. The limited pathology-detection performance observed even under these controlled conditions raises concerns about the model’s reliability in more challenging clinical settings, although this possibility was not directly evaluated. Prospective bedside studies of task-specific AI systems address a different level of evidence. For example, Fisher et al. [27] evaluated AI-enhanced focused cardiac ultrasound performed by noncardiologist physicians in emergency department and internal medicine settings and reported clinically relevant findings and subsequent management changes. That study involved dedicated algorithms integrated into patient examinations, and its findings cannot be directly extrapolated to a general-purpose M-LLM interpreting isolated frames. Educational use also requires caution. In a randomized ultrasonography training study, Hong et al. [28] found that a faculty-guided AI strategy improved short-term examination performance, but the benefit reflected the combined educational intervention, and ChatGPT-4o itself showed limited accuracy on image-based questions. Taken together, these studies suggest that clinician-supervised education and anatomical description are reasonable areas for further investigation, while the safety and effectiveness of such applications remain to be demonstrated for the model evaluated here.

### Limitations

This study has several limitations. First, because the evaluation was performed on a static, standardized educational image set, data contamination cannot be fully excluded. The model may have been exposed during training to these materials or related derivative content, which could have inflated performance, particularly in view/plane recognition and basic anatomical description. Second, our analysis was limited to static US frames and did not capture key dynamic components of real-world POCUS interpretation, such as pleural sliding, cardiac motion, Doppler or compression assessment, sequential scanning, and iterative operator feedback. The selected images also do not represent the full range of image quality encountered during bedside examinations in emergency medicine. Therefore, these findings should not be directly generalized to prospective clinical settings or patient-level outcomes. Third, each image was assessed in a single run using the chat interface and default settings, and repeated-run variability was not evaluated. Subsequent changes to the model/interface prevented a comparable repeated-run assessment under the original testing conditions, limiting conclusions regarding output stability and reproducibility. Finally, the small anatomical subgroups (12–17 images per group) limit the precision of regional estimates, and nonsignificant differences should not be interpreted as evidence of equivalent performance. The lack of blinding to prompt condition may have introduced assessment bias, while the absence of human and alternative-model comparators precludes direct comparisons with clinical experts or other AI systems.

## 5. Conclusions

In this paired in silico evaluation, GPT-5.6 Sol showed substantial gains in view/plane identification and anatomical structure recognition on static POCUS images when limited acquisition metadata was provided through informed prompting. In contrast, performance in pathological finding detection remained considerably lower, and persistent false-negative and false-positive responses limited the reliability of pathological assessment. These findings demonstrate that improved orientation and anatomical recognition do not necessarily translate into reliable pathology detection.

For POCUS applications in emergency medicine, the findings support further investigation of clinician-supervised educational assistance, view/plane confirmation, and anatomical labeling, but do not establish their clinical safety or effectiveness. Further studies incorporating dynamic clips, repeated-run stability analyses, human comparators, and prospective multicenter validation are needed before considering use during bedside patient assessment.

## Figures and Tables

**Table 1:** Interrater agreement analysis between two independent raters.

	Uninformed		Informed	
Evaluation Parameter	Weighted κ (95% CI)	Agreement (%)	Weighted κ (95% CI)	Agreement (%)
View/plane accuracy	0.750 (0.624–0.863)	84.8	0.868 (0.657–1.000)	96.7
Identification of anatomical structures	0.943 (0.883–0.987)	95.7	1.000 (1.000–1.000)	100.0
Pathological finding detection	0.943 (0.889–0.989)	94.6	0.956 (0.912–0.989)	95.7

Note: Interrater reliability between the two raters was assessed using quadratic-weighted Cohen’s kappa (κ) for ordinal agreement and interpreted according to Landis and Koch [20], where κ = 0.61–0.80 indicates substantial agreement and κ = 0.81–1.00 indicates almost perfect agreement. Abbreviations: CI, confidence interval.

**Table 2:** Comparison of GPT-5.6 Sol model performance across evaluation domains in uninformed and informed conditions.

Evaluation Domain	Uninformed Condition	Informed Condition	*p*-Value *
View/Plane Identification (0–2)			
Score, median (IQR)	2 (1–2)	2 (2–2)	<0.001
Success Rate, *n* (%; 95% CI)	54 (58.7; 48.5–68.2)	81 (88.0; 79.8–93.2)	<0.001
Anatomical Structure Identification (0–2)			
Score, median (IQR)	1 (1–2) ^a,^**	2 (2–2)	<0.001
Success Rate, n (%; 95% CI)	44 (47.8; 37.9–57.9)	76 (82.6; 73.6–89.0)	<0.001
Pathological Finding Detection			
Score, median (IQR)	0 (0–2) ^a,b,^*	1 (0–2) ^a,b,^*	0.001
Success Rate, n (%; 95% CI)	33 (35.9; 26.8–46.1)	45 (48.9; 38.9–59.0)	0.002
TOTAL SCORE (0–6)	4 (2–5) *	5 (4–6) *	<0.001

Note: Data are presented as median with interquartile range (IQR) for ordinal scores (range 0–2 for domains; 0–6 for total score) and as frequency (n) and percentage (%) for Success Rate. Confidence intervals (CIs) for fully correct rates were calculated using the Wilson score method. Differences between the two study conditions (Uninformed vs. Informed) were assessed using the Wilcoxon signed-rank test for ordinal scores and McNemar’s test for Success Rates. Global differences across the three evaluation domains were assessed using the Friedman test. Success Rate: Proportion of cases with score = 2 (Fully Correct). ^a^ Difference with View/Plane Identification. ^b^ Difference with Anatomical Structure Identification. * *p* < 0.001 and ** *p* = 0.003.

**Table 3:** Distribution of total performance scores by anatomical region.

Anatomical Region (*n*)	Uninformed Condition Mean ± SD	Informed Condition Mean ± SD	Change
Thorax (12)	5.08 ± 0.90	5.25 ± 0.97	+0.17
Abdomen (17)	4.11 ± 2.00	4.71 ± 1.61	+0.60
Renal/Urinary (12)	3.92 ± 1.38	4.83 ± 1.27	+0.91
Cardiac (12)	3.92 ± 1.73	4.25 ± 1.71	+0.33
Others (15)	3.13 ± 2.13	5.13 ± 1.06	+2.00
Vascular (12)	2.83 ± 1.19	4.42 ± 1.88	+1.59
IVC/Aorta (12)	2.17 ± 1.95	3.83 ± 2.17	+1.66
*p*-value	0.002	0.528	

Note: Data are presented as mean ± standard deviation (SD). Total Score ranges from 0 to 6. Differences among the seven anatomical regions within each condition (Uninformed or Informed) were assessed using the Kruskal-Wallis test. Change: Represents the arithmetic difference in mean scores between the Informed and Uninformed conditions. Abbreviations: IVC, inferior vena cava.

**Table 4:** Performance of GPT-5.6 Sol on pathological finding detection categorized by case status (positive vs. negative).

Outcome	Uninformed Condition *n* (%)	Informed Condition *n* (%)	Change (Percentage Points)
Pathology-Positive Images (*n* = 58)			
True positive	21 (36.2)	27 (46.6)	+10.4
Partially correct	6 (10.3)	5 (8.6)	−1.7
False negative	31 (53.4)	26 (44.8)	−8.6
Pathology-Negative Images (*n* = 34)			
True negative	12 (35.3)	18 (52.9)	+17.6
Partially correct	3 (8.8)	0 (0.0)	−8.8
False positive	19 (55.9)	16 (47.1)	−8.8

Note: Data are presented as frequency (n) and percentage (%). True Positive: Correct identification and justification of pathology (Score 2). False Negative: Missed pathology or incorrect labeling (Score 0). True Negative: Correct recognition that no pathology was present (Score 2). False Positive: Identification of pathology when none was present (Score 0).

**Table 5:** Comparison of recent multimodal language model studies involving ultrasound image interpretation and contextual information.

Study (Year)	Model and Dataset	Input Strategy	Main Findings
Brin et al. [24] (2025, online)	GPT-4V; 230 radiological images, including US	Direct image interpretation	In the US subset, anatomical region accuracy was 60.9%, while pathology identification accuracy was 9.1%.
Yao et al. [22] (2025)	GPT-4o; 80 breast US cases	Image-only vs. image plus report-derived descriptors	Diagnostic accuracy increased from 58% to 70% with additional textual information.
ELsharif et al. [14] (2026)	Six Vision LLMs; nine fetal US images	Standard vs. expert-designed prompts	ChatGPT-4o median total score increased from 8 to 18 with custom prompting.
Chen et al. [15] (2026)	ChatGPT-5.4; 202 thyroid nodules	Image-only, features-only, image plus features	Accuracy increased from 73.8% with image-only input to 84.2% with combined input; features-only accuracy was also 84.2%.
Present study (2026)	GPT-5.6 Sol; 92 static POCUS images	Image-only vs. image plus probe-region code	Fully correct rates increased from 58.7% to 88.0% for view/plane identification, 47.8% to 82.6% for anatomy, and 35.9% to 48.9% for pathology.

Note: US, ultrasound; POCUS, point-of-care ultrasound; LLM, large language model. Brin et al. was first published online in August 2024 and subsequently assigned to a 2025 journal volume. Differences in anatomical scope, input information, evaluation criteria, and outcome definitions preclude direct quantitative comparisons across studies.

## Data Availability

The dataset generated and/or analyzed during the current study may be shared by the corresponding author on reasonable request. The original ultrasound images are not publicly available and are excluded from data sharing because they were obtained from copyrighted educational textbooks and are subject to copyright restrictions.

## Supplementary Materials

The following supporting information can be downloaded at: https://www.mdpi.com/article/10.3390/diagnostics16213647/s1, Figure S1: Representative static ultrasound images and model interpretations under uninformed and informed prompting conditions. References [10,12] are cited in the Supplementary Materials.

## Abbreviations

The following abbreviations are used in this manuscript:

Abbreviation	Definition
AI	Artificial intelligence
AI-CAD	Artificial intelligence-based computer-aided diagnosis
ANT	Anterior chest
BI-RADS	Breast Imaging Reporting and Data System
CI	Confidence interval
ECG	Electrocardiogram
FAST	Focused assessment with sonography in trauma
IQR	Interquartile range
IRB	Institutional Review Board
IVC	Inferior vena cava
JPEG	Joint Photographic Experts Group
LLM	Large language model
LLQ	Left lower quadrant
LUQ	Left upper quadrant
MAX	Mid-axillary line
M-LLM	Multimodal large language model
PDF	Portable Document Format
PLAX	Parasternal long-axis
POCUS	Point-of-care ultrasound
PSAX	Parasternal short-axis
PSL	Parasternal (left)
RLQ	Right lower quadrant
RUQ	Right upper quadrant
SC	Subcostal
SD	Standard deviation
STARD-AI	Standards for Reporting Diagnostic Accuracy Studies–Artificial Intelligence
SU	Suprapubic
TRIPOD-LLM	Transparent Reporting of a Multivariable Model for Individual Prognosis or Diagnosis–Large Language Model
US	Ultrasound

## Appendix A

**Table A1:** Standardized prompt framework used in the study, including an informed condition providing limited acquisition metadata (probe region code).

Component	Content/Instruction
System role (persona)	“You are a clinician experienced in point-of-care ultrasound (POCUS). Your task is to interpret static ultrasound images using standard sonographic anatomy and terminology, and to provide a brief, structured assessment based only on findings visible in the image.”
Intended use statement (implicit target user)	“This output is generated for academic evaluation of ultrasound image interpretation performance; it is not intended for real-time patient care decision support.”
Input template (user input)	“A static ultrasound image is provided below. Interpret it like a POCUS expert. Do not assume additional clinical context unless explicitly provided.”
	**[INSERT IMAGE HERE]**
Prompt condition A—Uninformed	“Based **only** on the image, provide a structured response including: (1) anatomical region and ultrasound view/plane (e.g., parasternal long axis, transverse, longitudinal), (2) key anatomical structures visible, (3) pathology present/absent (if present, name it and provide 1–2 image-based supporting findings).”
Prompt condition B—Informed	“You obtained a static ultrasound image with the following probe region code (limited acquisition metadata): [RUQ, LUQ, RLQ, LLQ, SU, SC, PSL, MAX, ANT]. No organ name or clinical/pathology context is provided. Based on the image and only the given probe region code, respond with: (1) most likely anatomical region and view/plane, (2) key anatomical structures visible, (3) pathology present/absent (if present, 1–2 image-based supporting findings).”
Constraints	“Do not infer measurements, Doppler findings, or dynamic signs not visible in the still image (e.g., pleural sliding). Do not provide differential diagnoses. Do not provide treatment/clinical management recommendations. Use standard ultrasound terminology. If image quality is insufficient, state this explicitly.”
Output format	“Structured, bullet-pointed response with headings (items 1–3).”
Abbreviations (probe region code labels):	RUQ: Right upper quadrant; LUQ: Left upper quadrant; RLQ: Right lower quadrant; LLQ: Left lower quadrant; SU: Suprapubic; SC: Subcostal; PSL: Parasternal (left); MAX: Mid-axillary line; ANT: Anterior chest.

Note: In the informed condition, the probe region code (limited acquisition metadata) was the only extra cue provided; it was restricted to the predefined list above and did not include organ names or any clinical/pathology context.

## Appendix B

**Table A2:** Key anatomical structures expected to be visible in the reference ultrasound views according to anatomical regions.

Anatomical Region	Ultrasound View/Plane	Key Anatomical Structures Expected to Be Visible	Note
Cardiac	Parasternal long-axis (PLAX) cardiac view	Left ventricle, left atrium, mitral valve level, aortic root, left ventricular outflow tract, the long-axis portion of the interventricular septum, myocardial walls, pericardial contour	The aortic root and valvular level may be only partially discernible on a static frame.
Cardiac	Parasternal short-axis (PSAX) cardiac view	Short-axis view of the left ventricle, myocardial ring, papillary muscles, adjacent portion of the right ventricle, pericardial contour	The exact imaging level may not always be definitively distinguishable on a single static frame.
Cardiac	Apical four-chamber cardiac view	Left ventricle, right ventricle, left atrium, right atrium, interventricular septum, mitral valve plane, tricuspid valve plane, apex	Valvular and septal details may have variable clarity on a static frame.
Cardiac	Subcostal cardiac view	Liver parenchyma, right atrium, right ventricle, left atrium, left ventricle, septal structures, myocardium, pericardial contour	Similar subcostal cardiac windows were grouped under the same heading.
Thorax	Pleural–lung view	Superficial chest wall tissues, ribs, posterior acoustic shadowing, pleural line, subpleural area, diaphragm, basal lung parenchyma, adjacent solid organ parenchyma	Similar pleural and basal lung windows were evaluated within the same group.
Thorax	Lung M-mode view	Superficial chest wall tissues, pleural line, subpleural artifact patterns	Dynamic interpretation is outside the scope of this document.
Abdomen	Right upper quadrant FAST view	Liver parenchyma, right kidney, hepatorenal recess, diaphragm	The key structures expected in the hepatorenal FAST window were used as the reference.
Abdomen	Left upper quadrant FAST view	Spleen, left kidney, splenorenal recess, diaphragm	The key structures expected in the splenorenal FAST window were used as the reference.
Abdomen	Hepatobiliary vascular view	Liver parenchyma, portal vein, inferior vena cava, adjacent vascular or tubular structures at the level of the porta hepatis, gallbladder	Detailed differentiation of vascular and tubular structures may be limited without Doppler imaging.
Renal–Urinary	Kidney view	Renal contour, renal parenchyma, corticomedullary differentiation, renal sinus, pelvis–collecting system region	Similar renal windows were grouped under a single heading.
Renal–Urinary	Bladder view	Bladder lumen, bladder wall, posterior acoustic enhancement, surrounding pelvic soft tissues	Transverse and longitudinal bladder windows were grouped together.
Vascular	Cervical vascular view	Common carotid artery, internal jugular vein, vessel walls, surrounding soft tissues	Similar cervical vascular structures were evaluated under a single heading.
Vascular	Peripheral vascular view	Arterial and/or venous lumen, vessel walls, skin-subcutaneous tissue, muscle-fascial planes	Femoral, popliteal, and similar peripheral vascular windows were grouped within this category.
IVC/Aorta	Abdominal aortic view	Abdominal aortic lumen, aortic wall, vertebral body or posterior vertebral landmark, surrounding retroperitoneal soft tissues	The vertebral landmark may not always be clearly visible on every static frame.
IVC/Aorta	Inferior vena cava view	Inferior vena cava lumen, IVC walls, adjacent liver parenchyma, adjacent aorta, surrounding soft tissue planes	IVC views obtained in different imaging axes were grouped together based on similar anatomical expectations.
Other	Airway / laryngeal view	Trachea, air-mucosal interface, tracheal cartilaginous structures, thyroid cartilage level, linear structures corresponding to the vocal cords, surrounding soft tissues	Similar anterior neck windows were grouped under the same heading.
Other	Ocular view	Globe contour, vitreous chamber, posterior globe wall, optic nerve, anterior globe boundary	The posterior globe wall and optic nerve complex may show variable clarity on static images.
Other	Scrotal view	Testicular parenchyma, testicular contour or capsule, homogeneous internal echotexture, part of the adjacent epididymal structures	Longitudinal and transverse scrotal windows were grouped together.

Note: For each ultrasound view/plane, the key anatomical structures expected to be visible on a single static frame are listed. The purpose is to clearly and systematically present the anatomical reference standard used during the scoring of static ultrasound images. The listed structures represent the principal sonographic anatomical landmarks expected to be identifiable in the relevant view. Minor adjacent structures that cannot be reliably distinguished on a static image were not considered mandatory for full anatomical correctness. Abbreviations: FAST, focused assessment with sonography in trauma; IVC, inferior vena cava; PLAX, parasternal long-axis; PSAX, parasternal short-axis.

## References

[1] Kung T.H., Cheatham M., Medenilla A., Sillos C., De Leon L., Elepaño C., Madriaga M., Aggabao R., Diaz-Candido G., Maningo J. (2023). Performance of ChatGPT on USMLE: Potential for AI-assisted medical education using large language models. PLoS Digit. Health.

[2] Ayers J.W., Poliak A., Dredze M., Leas E.C., Zhu Z., Kelley J.B., Faix D.J., Goodman A.M., Longhurst C.A., Hogarth M. (2023). Comparing physician and artificial intelligence chatbot responses to patient questions posted to a public social media forum. JAMA Intern. Med..

[3] Bhayana R., Krishna S., Bleakney R.R. (2023). Performance of ChatGPT on a radiology board-style examination: Insights into current strengths and limitations. Radiology.

[4] AlSaad R., Abd-alrazaq A., Boughorbel S., Ahmed A., Renault M.A., Damseh R., Sheikh J. (2024). Multimodal large language models in health care: Applications, challenges, and future outlook. J. Med. Internet Res..

[5] Altinbilek E., Az A., Sogut O., Dogan Y., Akdemir T., Belen E., Biter H.I., Saricicek T., Ozcomlekci M., Kilic N. (2025). Task-specific versus general-purpose AI models in ECG analysis: A comparative study with emergency medicine specialists. Am. J. Emerg. Med..

[6] Gobira M., Nakayama L.F., Moreira R., Andrade E., Regatieri C.V.S., Belfort R. (2023). Performance of ChatGPT-4 in answering questions from the Brazilian National Examination for Medical Degree Revalidation. Rev. Assoc. Med. Bras..

[7] Lai U.H., Wu K.S., Hsu T.Y., Kan J.K.C. (2023). Evaluating the performance of ChatGPT-4 on the United Kingdom Medical Licensing Assessment. Front. Med..

[8] Umit T.B., Arslan M., Inan G., Fettahoglu S.T., Yildiz A., Sarbay I., Taskan H., Biter H.I., Sogut O. (2026). Can ChatGPT compete with emergency medicine specialists? A two-stage assessment of ECG interpretation supported by clinical information. BMC Emerg. Med..

[9] Dogan Y., Az A., Sogut O., Akdemir T., Ergenc H., Umit T.B., Armagan B.N., Ozturk M.F. (2026). Domain-specific and computer-vision-driven versus general-purpose AI models in PA-CXR analysis: A comparative study with emergency-medicine specialists. J. Imaging Inform. Med..

[10] Robba C., Messina A., Wong A., Vieillard-Baron A. (2023). Basic Ultrasound Skills “Head to Toe” for General Intensivists.

[11] Soliman-Aboumarie H., Miglioranza M.H., Gargani L., Volpicelli G. (2023). Cardiopulmonary Point of Care Ultrasound.

[12] Gottlieb M., Panebianco N. (2025). Advanced Point-of-Care Ultrasound: A Comprehensive Review.

[13] Chen Z., Ye M., Liang J., Chen F., Ying M.T.C. (2026). ChatGPT-5-based large language model analysis versus an FDA-approved AI-CAD system for thyroid nodule ultrasound evaluation. Eur. J. Radiol..

[14] ELsharif W., Alzubaidi M., Tukur M., Magram A., Anver F., Hamza A., Said S., Khan R., Househ M., Agus M. (2026). Benchmarking Vision LLMs in fetal ultrasound interpretation: A five-point expert evaluation of standard vs. custom prompts. Biomed. Eng. Online.

[15] Chen Z., Chen F., Wang Y. (2026). Impact of clinician-recorded ACR TI-RADS features on ChatGPT-5.4-based thyroid nodule classification. Semin. Ultrasound CT MRI.

[16] Zou M., Xiao M., Zhu Q., Zhang J., Li J., Lv K. (2026). Multimodal large language models for breast ultrasound report auditing: Workflow-error detection, false-positive burden, and limits of key-image interpretation. J. Imaging Inform. Med..

[17] Altintas Mese C., Mese I., Akinci D’Antonoli T. (2026). Zero-shot performance of a general-purpose vision-language model for pediatric appendicitis diagnosis. Pediatr. Radiol..

[18] Sounderajah V., Guni A., Liu X., Collins G.S., Karthikesalingam A., Markar S.R., Golub R.M., Denniston A.K., Shetty S., Moher D. (2025). The STARD-AI reporting guideline for diagnostic accuracy studies using artificial intelligence. Nat. Med..

[19] Gallifant J., Afshar M., Ameen S., Aphinyanaphongs Y., Chen S., Cacciamani G., Demner-Fushman D., Dligach D., Daneshjou R., Fernandes C. (2025). The TRIPOD-LLM reporting guideline for studies using large language models. Nat. Med..

[20] Landis J.R., Koch G.G. (1977). The measurement of observer agreement for categorical data. Biometrics.

[21] Miaojiao S., Xia L., Xian Tao Z., Zhi Liang H., Sheng C., Songsong W. (2025). Using a large language model for Breast Imaging Reporting and Data System classification and malignancy prediction to enhance breast ultrasound diagnosis: Retrospective study. JMIR Med. Inform..

[22] Yao J.Q., Zhang R., Yang Z.B., Zhang B., Jiang S.Q., Jiang L., Zhang X.E., Xie X.Y., Huang T.Y., Xu M. (2025). Multimodal large language models in ultrasound diagnosis of breast masses: A multicenter comparative analysis based on GPT-4o, radiologists, and convolutional neural network (CNN). Quant. Imaging Med. Surg..

[23] Lo C.M., Sung S.F. (2025). Recognizing artery segments on carotid ultrasonography using embedding concatenation of deep image and vision-language models. Phys. Med. Biol..

[24] Brin D., Sorin V., Barash Y., Konen E., Glicksberg B.S., Nadkarni G.N., Klang E. (2025). Assessing GPT-4 multimodal performance in radiological image analysis. Eur. Radiol..

[25] Chen Z., Chambara N., Wu C., Lo X., Liu S.Y.W., Gunda S.T., Han X., Qu J., Chen F., Ying M.T.C. (2025). Assessing the feasibility of ChatGPT-4o and Claude 3-Opus in thyroid nodule classification based on ultrasound images. Endocrine.

[26] Koga S., Du W. (2025). From text to image: Challenges in integrating vision into ChatGPT for medical image interpretation. Neural Regen. Res..

[27] Fisher L., Fiman M., Yarkoni Y., Segal E., Fishman B., Faierstein K., Rubin N., Am-Shalom A., Amital H., Zhao Q. (2026). Artificial intelligence-enhanced cardiac point-of-care ultrasound: A prospective single-arm study. Mayo Clin. Proc. Digit. Health.

[28] Hong D.R., Huang C.Y., Gao J. (2026). ChatGPT-4o with faculty guidance outperforms AI-only and traditional learning in ultrasonography training: A randomized trial. Front. Digit. Health.

