# Supplementary material for: From Anatomy to Pathology: How Well Can AI Interpret Static Point-of-Care Ultrasound Images?
Source: Diagnostics (Basel). 2026 Nov 4;16(21):3647. doi: 10.3390/diagnostics16213647 (PMC13762227; doi:10.3390/diagnostics16213647)
Supplement: Supplementary file 1 [file diagnostics-16-03647-s001.zip › diagnostics-4661156-supplementary.pdf]

Supplementary material

From Anatomy to Pathology: How Well Can AI Interpret Static Point-of-Care Ultrasound Images?

Figure S1. Representative static ultrasound images and model interpretations under uninformed and informed prompting conditions. Interpretations are English summaries of the recorded responses, not verbatim quotations. Scores are reproduced from the scoring record. These illustrative cases do not summarize performance across the full dataset.

Panel A Vitreous hemorrhage

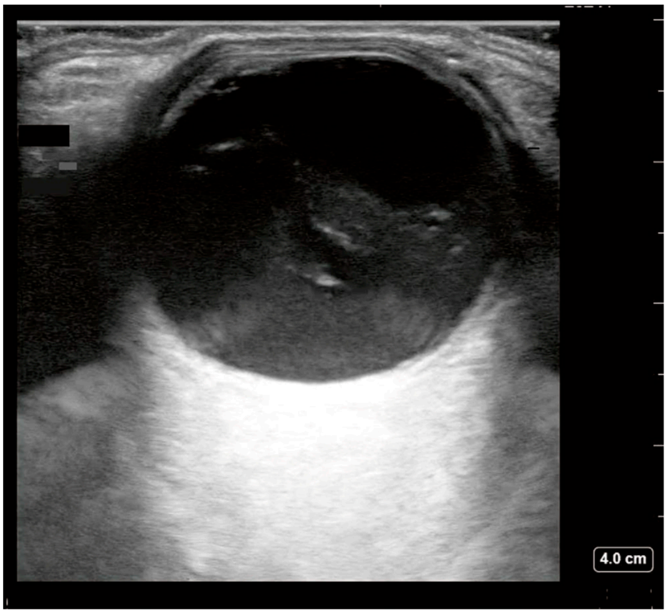

Reference finding: Vitreous hemorrhage. Dataset image 61.

Uninformed condition: The image was interpreted as a superficial complex cystic lesion or fluid collection containing internal debris. The ocular globe was not identified.

Informed condition: The ocular globe and vitreous cavity were identified. Internal vitreous echoes or debris were described; a definite retinal or choroidal detachment was not identified.

Scoring domain	Uninformed	Informed
View or plane identification	1	2
Anatomical structure identification	0	2
Pathology interpretation	1	2
**Total score**	**2**	**6**

Each domain was scored from 0 to 2; the total score ranged from 0 to 6.

Recognition of the anatomical region improved after acquisition information was provided. Pathology interpretation received a full score in the scoring record.

Image credit: [12] Kim DJ, Sheppard G, Thavanathan R. Ocular Ultrasound. In: Gottlieb M, Panebianco N, editors. Advanced Point-of-Care Ultrasound: A Comprehensive Review. Springer; 2025. Figure 15.5, p. 298. https://doi.org/10.1007/978-3-031-85308-1_15. Reproduction permission pending. For editorial review only; not for publication.

Figure S1 continued

Panel B Cholelithiasis

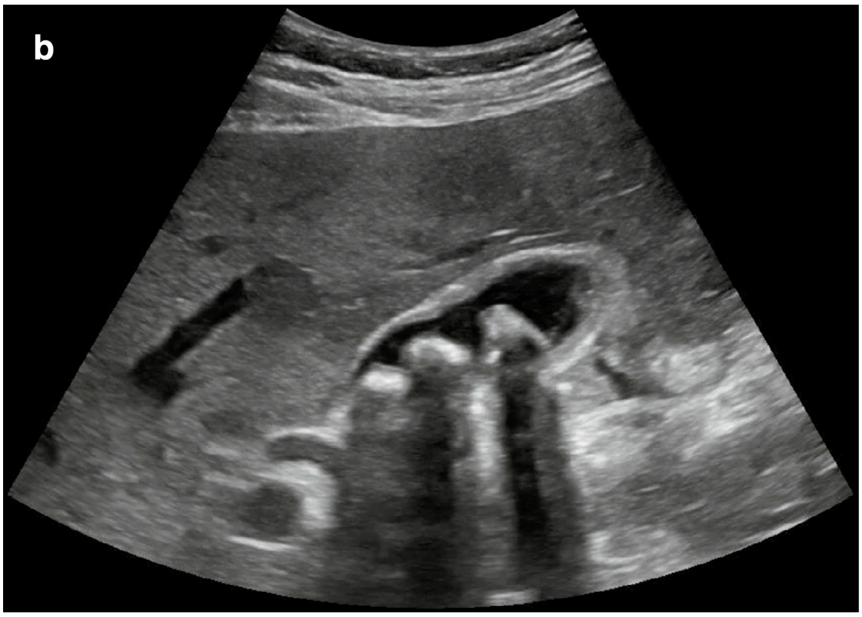

Reference finding: Cholelithiasis. Dataset image 58.

Uninformed condition: Multiple echogenic foci with posterior acoustic shadowing were identified within a structure considered to be the gallbladder, and cholelithiasis was reported.

Informed condition: The gallbladder was identified in the right upper quadrant, and multiple gallstones with posterior acoustic shadowing were reported.

**Scoring domain**	**Uninformed**	**Informed**
View or plane identification	2	2
Anatomical structure identification	2	2
Pathology interpretation	2	2
**Total score**	**6**	**6**

Each domain was scored from 0 to 2; the total score ranged from 0 to 6.

The reference pathology was correctly identified under both prompting conditions.

Image credit: [12] Zwank MD, Leo MM, Schutzer CM. Hepatobiliary, Pancreas, and Spleen Ultrasound. In: Gottlieb M, Panebianco N, editors. Advanced Point-of-Care Ultrasound: A Comprehensive Review. Springer; 2025. Figure 10.24b, p. 179. Original image courtesy of Michael Gottlieb, MD. https://doi.org/10.1007/978-3-031-85308-1_10. Reproduction permission pending. For editorial review only; not for publication.

Figure S1 continued

Panel C Right ventricular dilatation

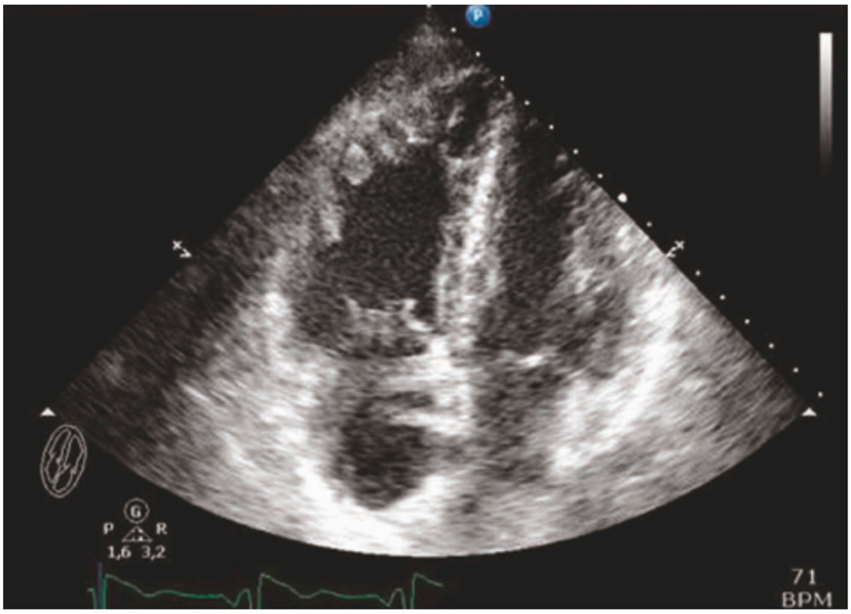

Reference finding: Right ventricular dilatation. Dataset image 45.

Uninformed condition: The apical four-chamber view and the cardiac chambers were identified. An anechoic area around the heart was interpreted as pericardial effusion.

Informed condition: The apical four-chamber view and the cardiac chambers were again identified. Pericardial effusion was reported, while right ventricular dilatation was not identified.

**Scoring domain**	**Uninformed**	**Informed**
View or plane identification	2	2
Anatomical structure identification	2	2
Pathology interpretation	0	0
**Total score**	**4**	**4**

Each domain was scored from 0 to 2; the total score ranged from 0 to 6.

View and anatomy identification received full scores in both conditions, whereas pathology interpretation received a score of zero. The source caption describes acute cor pulmonale in acute respiratory distress syndrome, with a right-to-left ventricular diameter ratio greater than one.

Image credit: [10] Pisani L, Smit MR, Tuinman PR. In: Robba C, Messina A, Wong A, Vieillard-Baron A, editors. Basic Ultrasound Skills "Head to Toe" for General Intensivists. Springer; 2023. Chapter 24, Figure 24.6, p. 400. https://doi.org/10.1007/978-3-031-32462-8_24. Reproduction permission pending. For editorial review only; not for publication.
